# Supragastric belching in Japan: lower prevalence and relevance for management of gastroesophageal reflux disease compared to United Kingdom

**DOI:** 10.1007/s00535-020-01720-9

**Published:** 2020-08-24

**Authors:** Akinari Sawada, Hideaki Itami, Kenichiro Nakagawa, Shinji Hirano, Hiroyuki Kitamura, Rieko Nakata, Shingo Takashima, Yasuaki Abe, Masahiro Saito, Etsuro Yazaki, Osamu Kawamura, Fumio Tanaka, Toshihisa Takeuchi, Tomoyuki Koike, Atsushi Masamune, Yasuhiro Fujiwara, Kazuhide Higuchi, Daniel Sifrim

**Affiliations:** 1 Barts and The London School of Medicine and Dentistry, Wingate Institute of Neurogastroenterology, Blizard Institute, Upper GI Physiology Unit Royal London Hospital, Queen Mary University of London, 26 Ashfield Street, London, E12AJ UK; 2 Department of Gastroenterology, Osaka City University Graduate School of Medicine, Osaka, Japan; 3 Division of Gastroenterology, Tohoku University Graduate School of Medicine, Sendai, Japan; 4 Department of Gastroenterology, Kamimoku SPA Hospital, Minakami, Japan; 5 Second Department of Internal Medicine, Osaka Medical College, Takatsuki, Japan

**Keywords:** Supragastric belching, Gastroesophageal reflux disease, Impedance-pH monitoring, Cross-cultural study

## Abstract

**Background:**

Supragastric belching (SGB) may play a role in the pathophysiology of proton pump inhibitors (PPIs)-refractoriness in gastroesophageal reflux disease (GERD). SGB may be present in up to 40% of reflux symptoms in PPI-refractory GERD. Most reports on SGB have come from Western countries, and little is known about the prevalence and relevance of SGB in Asian refractory GERD patients. This study aimed at comparing the role of SGB in GERD patients in Japan and the UK.

**Methods:**

We re-analyzed impedance-pH monitoring tracings from patients who were referred to tertiary centers in Japan and the UK due to PPI-refractory reflux symptoms. The prevalence of excessive SGB and the impact of SGB on reflux symptoms were compared between the two countries.

**Results:**

Impedance-pH tracings from124 Japanese and 83 British patients were re-analyzed. Japanese patients were significantly younger and had smaller body mass index than the British (*P* < 0.001). Japanese patients had significantly lower prevalence of excessive SGB (18.5%) than the UK (36.1%) irrespective of reflux phenotype (*P* = 0.006). Logistic regression analysis showed that the geographical/cultural difference was the only factor associated with the different prevalence of SGB (odds ratio; 2.91, 95% CI 1.09–7.73, *P* = 0.032). SGB were related to typical reflux symptoms very rarely in Japan [0% (0–4.9)] compared to the UK [35% (0–54.1)] (*P* = 0.071).

**Conclusions:**

The prevalence of SGB and their impact on reflux symptoms is significantly lower in Japan compared to the UK. The difference is not related to reflux parameters but might come from ethnic/cultural factors to be further characterized.

## Introduction

Belching is defined as “audible escape of air from the esophagus or the stomach into the pharynx” [1]. Although it is a common phenomenon, it can impair quality of life when excessive [2]. Belching can be divided into two types by impedance monitoring: gastric belching and supragastric belching (SGB). Gastric belching is a physiological mechanism to vent swallowed air from the stomach, while SGB is a behavior by which air is swallowed or sucked down into the esophagus and subsequently expelled through the pharynx [3].

Several studies reported the prevalence of increased belching in the general population ranging from 9.3 to 28.8%, and is much higher among patients having reflux symptoms [4–8]. These studies included both types of belching, however, the prevalence of SGB might differ regionally as it is the case with gastroesophageal reflux disease (GERD) [9].

SGB has been increasingly recognized as a hidden culprit of proton pump inhibitor (PPI)-refractoriness in some patients with GERD. Many of these patients often describe typical reflux symptoms (heartburn or regurgitation) rather than belching even though a SGB was the initial cause of a symptomatic reflux event [10, 11]. A recent study from our group showed that 35% of PPI-refractory reflux patients at the Royal London Hospital, UK, had excessive SGB frequently associated with reflux symptoms [11, 12]. Proper identification of SGB is critical for therapeutic management because this type of belching rarely responds to acid suppression therapy or pain modulators. Alternatively, patients with excessive SGB require a specific psychological approach [10].

Most studies about SGB report data from Western population and there are few studies from Asian [13, 14]. It is known that visceral pain perception differs among races and countries [15]. We hypothesized that the prevalence, perception and/or relationship between SGB and reflux symptoms could differ between cultures.

The aim of this study was (i) to assess the prevalence of excessive SGB in Japanese patients with GERD and (ii) to compare the prevalence and relevance of SGB for reflux symptoms generation between Japanese and British patients.

## Methods

### Study subjects

#### Healthy subjects

24-h impedance-pH monitoring of available 17 Japanese healthy volunteers (HVs) were re-analyzed [16] and the prevalence of SGB was compared with data published from 40 healthy subjects in the UK [17]. Healthy subjects did not have gastro-intestinal (GI) symptoms or history of upper GI surgery. They were recruited by advertisement.

#### Patients with GERD

We included patients with typical reflux symptoms (heartburn, regurgitation and/or chest pain) referred to tertiary referral centers for reflux monitoring with impedance-pHmetry (Osaka City University Hospital or Tohoku University Hospital in Japan, Royal London Hospital in the UK). These patients underwent on-PPI (Japan and the UK) or potassium-competitive acid blockers (P-CAB) (Japan) impedance-pH monitoring for assessment of PPI-refractory esophageal reflux symptoms which were defined as persistence of symptoms despite the standard clinical dose of PPI/PCAB treatment for more than 8 weeks. Patients were excluded if (i) they were younger than 20 years of age or (ii) had belching as a main symptom (we focused on the role of SGB in patients with predominant reflux symptoms). We interrogated the database to collect patients’ clinical information including endoscopic findings.

The institutional review board at the Osaka City University and Tohoku University approved the study. Also, we obtained approval from Quality and Service Improvement department at the Royal London Hospital, UK. This study was carried out according to the ethical principles of the Declaration of Helsinki.

#### Esophageal impedance-pH monitoring

In both countries, impedance pH monitoring [Sandhill Scientific, Highlands Ranch, CO, USA (Japan and the UK) or OMOM System, Jinshan Science and Technology, Chongqing, China (the UK)] was performed “on”-PPIs/P-CAB after overnight fasting.

A preceding stationary manometry located the position of lower esophageal sphincter (LES). The impedance-pH catheter was inserted so that the esophageal pH sensor was located 5 cm above the LES, and 6 impedance channels were located 3, 5, 7, 9, 15 and 17 cm above the LES respectively. The placement was confirmed radiologically if manometry was unavailable (43 Japanese patients) so that a radio-opaque esophageal pH sensor was located 5 cm above the crural diaphragm where domes of the both diaphragms meet on a vertebra taking into account the size of hiatus hernia measured in endoscopy. The data were stored in a portable recorder. During the recording period, subjects were encouraged to continue with their usual daily activities and meals. Patients logged the time when feeling a particular reflux symptom during the test by pressing a button on the recorder.

### Data analysis

#### 24-h impedance-pH monitoring

All the impedance-pH monitoring tracings were edited manually and re-analyzed for this study as previously described [18]. In brief, reflux was defined as retrograde impedance drop by at least 50% from baseline in at least the two most distal channels. Acid reflux were defined as (i) reflux with pH drop to < 4 or (ii) reflux with maintaining pH < 4 if pH was already < 4 beforehand. Non-acidic reflux was defined as reflux with pH > 4. Proximal extent of reflux was defined as reflux reaching 15 cm above the LES. Esophageal acid exposure time was calculated as the percentage of time with esophageal pH < 4 during the total 24 hr recording. Pathological acid exposure time (AET) “on” PPIs/P-CAB was defined as > 1.6% [19]. Besides, percentage of gastric pH < 4 was assessed to characterize the extent of acid suppression by PPIs/P-CAB.

#### Definition of Supragastric belching

SGB was identified in impedance-pH monitoring using the definition by Bredenoord et al. [3] as aboral movement of rapid impedance increase (> 1000 ohms), followed by a return to baseline in the retrograde way. More than 13 SGBs/24 hr was considered as excessive SGB based on our previous study in healthy subjects [17]. SGB were classified into three patterns based on their time relationship with reflux events as follows: (i) SGB-induced reflux (i.e., SGB followed by reflux within one second), (ii) SGB during reflux or (iii) SGB without reflux.

#### Reflux symptom association

Symptom index (SI) [20] and Symptom association probability (SAP) [21] assessed reflux symptom association for typical esophageal reflux symptom (i.e., heartburn, regurgitation and/or chest pain). In brief, SI indicates the proportion of reflux-related symptoms (i.e., symptom marked within 2 min from the onset of a reflux) to the total number of symptoms. SAP is the probability of reflux symptom association calculated by Fisher’s exact test where checking whether consecutive every 2 min period includes symptom and/or reflux. Symptom reflux association was considered as positive when either SI was ≥ 50% or SAP was > 95%.

#### Reflux phenotypes in patients with typical symptoms studied on-PPIs/P-CAB

On the basis of AET and reflux symptom association, patients without esophagitis were divided into the following three phenotypes: (i) non-erosive reflux disease (NERD) (AET > 1.6%), (ii) reflux hypersensitivity (AET < 1.6% and positive reflux symptom association) or (iii) functional heartburn (AET < 1.6% and negative reflux symptom association).

#### Association between Supragastric belching and reflux symptoms

A reflux symptom was considered as associated with a SGB when logged by a patient within 20 s after the SGB as previously described [12].

### Statistical analysis

Continuous and categorical variables were expressed as mean ± standard deviation or median (interquartile) and numbers (percent) respectively. Categorical variables were compared between groups using the Chi square test, except for variables with small numbers in some categories where Fisher’s exact test was preferred. Continuous variables were compared between groups using the unpaired *t* test for variables found to follow a normal distribution, or the Mann–Whitney test otherwise.

To evaluate the difference of excessive SGB in the two countries, firstly, we performed univariate analyses comparing the characteristics of the two patient groups. Secondly, adjustments were made for factors found to show some differences between groups from the initial analyses. Due to the binary nature of the outcome (excessive SGB), the analysis was performed using logistic regression. To restrict the number of variables in the model, only variables showing some evidence of a difference between countries (*P* < 0.2) were adjusted for such as age, body mass index (log scale), study indication, total AET (log scale), total reflux episodes (log scale), acid reflux episodes (log scale), proximal extent (log scale), gastric pH < 4 holding time and reflux symptom association. Due to the different kinds or standard dose of PPIs between the two countries, gastric pH < 4 holding time was included in the adjustment as above. All analyses were performed using R software, version 3.3.1 (R Core Team, Vienna, Austria). *P* value < 0.05 was considered statistically significant.

## Results

### Healthy subjects

From the 17 Japanese HVs, 2 subjects were excluded due to pathological acid exposure (AET > 4%). In the remaining 15 subjects (mean age 35, 12 males), the median number of SGBs were 1 (0–3)/24 hr which did not differ from the British HVs (mean age 36, 20 males) (0 (0–4)/24 hr, P = 0.951. The 95th percentile value: 13/24 hr). Besides, the 95th percentile of SGB in Japanese HVs was 12.8. Consequently, we adopted the same cut-off value for excessive SGB (> 13/24 h) for further analysis in Japanese patients.

GERD patients (Table 1).

**Table 1:** Demographic and clinical characteristics of all the patients in Japan and the UK

	Japan (*N* = 124)	UK (*N* = 83)	*P* value
Age (y)	58.7 ± 15.7	46.2 ± 14.1	< 0.001
Female (n, %)	73 (58.9%)	45 (54.2%)	0.567
BMI (kg/m^2^)	21.3 (19.1–23.4)	26.1 (23.0–29.9)	< 0.001
Mental disorders (n, %)	8 (6.5%)	7 (8.4%)	0.591
Study indication			
Esophageal symptoms (*n*, %)			0.075
Only heartburn	51 (41.1%)	29 (34.9%)	
Only regurgitation	11 (8.9%)	12 (14.5%)	
Only chest pain	2 (1.6%)	7 (8.4%)	
Heartburn and regurgitation	42 (33.9%)	22 (26.5%)	
Regurgitation and chest pain	2 (1.6%)	4 (4.8%)	
Heartburn and chest pain	8 (6.5%)	2 (2.4%)	
All three symptoms	8 (6.5%)	6 (7.2%)	
Extra-esophageal symptoms (*n*, %)	60 (48.4%)	42 (50.6%)	0.778

Extra-esophageal symptoms include throat discomfort, cough, dysphagia, abdominal discomfort or belching

*BMI* body mass index

From the Japanese data, 5 patients were excluded due to technical recording problems leaving 124 patients for analysis.

From the British data, 16 patients were excluded due technical recording problems or belching being the main symptom leaving 83 patients for analysis.

The Japanese patients were significantly older (*P* < 0.001) and had lower BMI (*P* < 0.001) than the British patients. There was no difference in study indication (esophageal and extra-esophageal symptoms) between Japan and the UK. The proportion of patients with abnormal psychiatric background did not differ between the two countries (4 depression, 3 anxiety disorder, and 1 Post-traumatic stress disorder (PTSD) in Japan, 4 depression, 1 conversion disorder, 1 anxiety disorder and 1 PTSD in the UK).

### Patients characteristics in Japan and the UK

Table 2 shows results of impedance-pH monitoring in the two countries. Although both groups were studied “on” PPIs/P-CAB, the Japanese patients had stronger inhibition of gastric acid secretion than the British patients (*P* < 0.001). As a consequence, patients in Japan had lower esophageal acid exposure and number of acid reflux episodes than in the UK.

**Table 2:** Measurements of on-PPIs/P-CAB Impedance-pH monitoring of patients in Japan and the UK

	Japan (*N* = 124)	UK (*N* = 83)	*P* value
Impedance-pH monitoring			
Acid exposure time (%)	0.2 (0–0.9)	2.0 (0.3–8.3)	< 0.001
Number of reflux episodes (*n*)			
Total	43 (20–59)	47 (30–85)	0.030
Acid	3 (0–9)	16 (6–32)	< 0.001
Non-acid	30 (16–49)	26 (13–44)	0.280
Proximal extent	11 (4–26)	20 (8–34)	0.003
Gastric pH < 4 holding time (%)	33.6 (12.5–59.0)	62.3 (43.2–78.5)	< 0.001
Reflux symptom association positive	49 (39.5%)	41 (49.4%)	0.198

*PPIs* proton pump inhibitors, *P-CAB* potassium-competitive acid blockers, *SI* symptom index, *SAP* symptom association probability

Regarding reflux phenotypes, functional heartburn (FH) was predominant in Japan [*n* = 55 (44%)], followed by reflux hypersensitivity (RH) [*n* = 37 (30%)], non-erosive reflux disease (NERD) (*n* = 20 (16%)) and esophagitis [*n* = 12 (10%)], whereas NERD comprised almost half of the British patients [*n* = 43 (52%)] and the remaining were RH [*n* = 16 (19%)], FH [*n* = 24 (29%)] and no esophagitis. Distribution of phenotypes were significantly different between the two countries (*P* < 0.001).

**Fig. 1:**
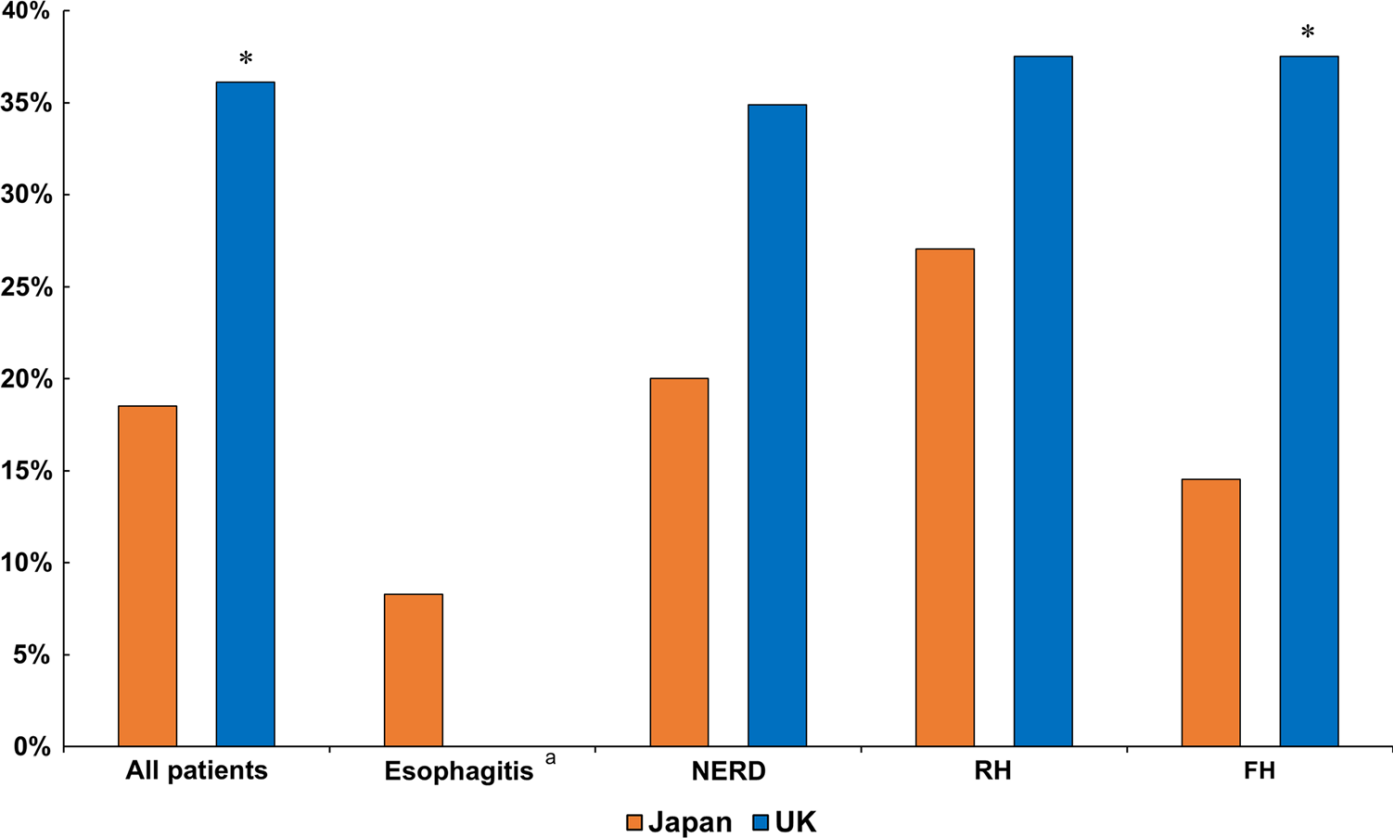
Prevalence of excessive SGB in each reflux phenotype in the two countries. *SGB* supragastric belching, *NERD* non-erosive reflux disease, *RH* reflux hypersensitivity, *FH* functional heartburn. ^a^No patients with esophagitis in the UK, *P < 0.05 compared to Japan

The prevalence of excessive SGB in the Japanese patients (18.5%) was significantly lower than that found in the British patients. (36.1%) (*P* = 0.006) irrespective of phenotype (Fig. 1). Interestingly, when we looked at the total number of SGB found in these patients (with excessive SGB) there were no difference between both countries [36 (20–71)/24 hr for Japan, 35 (24–80)/24 hr for the UK, *P* = 0.760] (Table 3). In Japanese patients, no difference was found between on-PPI patients (*n* = 110) and on-PCAB patients (*n* = 14) in the prevalence of excessive SGBs (18.2% for on-PPI, 21.4% for on-PCAB, *P* = 0.723).

**Table 3:** Characteristics of excessive SGB in patients with reflux symptom in the two countries

	Japan (*N* = 23)	UK (*N* = 30)	*P* value
Total number of SGBs (*n*)	36 (20–71)	35 (24–80)	0.760
SGB-induced reflux (*n*)	6 (2–10)	5 (3–16)	0.373
SGB during reflux (*n*)	7 (5–16)	14 (4–33)	0.218
SGB without reflux (*n*)	19 (8–39)	15 (9–34)	0.943
Proportion of SGB-induced reflux to All reflux (%)	10.3 (4.0–24.3)	11.9 (5.3–36.1)	0.760

*SGB* supragastric belching

In order to understand possible factors that could explain the differences in the prevalence of SGB between Japan and the UK, we performed a logistic regression analysis that showed adjusted odds ratio (odds of excessive SGB in the UK relative to odds in Japan) of 2.91 (95% CI, 1.09–7.73; *P* = 0.032) (Table 4). This analysis showed that the lower prevalence of excessive SGB in Japanese patients was more related to regional difference rather than clinical characteristics (age, body mass index and symptoms), reflux profile (total AET, total and acid reflux episodes, proximal extent), reflux symptom association and the extent of acid suppression (gastric pH < 4 holding time).

**Table 4:** Logistic regression analysis comparing excessive SGB between Japan and the UK

Adjustments	Odds Ratio^a^ (95% CI)	*P* value
Unadjusted	2.49 (1.31–4.70)	< 0.01
Adjusted^b^	2.91 (1.09–7.73)	0.032

*SGB* supragastric belching, *CI* confidence interval, *BMI* body mass index, *AET* acid exposure time

^a^Odds ratio reported as odds of SGB > 13 in the UK relative to odds in Japan

^b^Adjusted for age, BMI (log scale), study indication, total AET (log scale), total reflux episodes (log scale), acid reflux episodes (log scale), proximal extent (log scale), gastric pH < 4 holding time and reflux symptom association

### Impact of Supragastric belching on acid reflux in Japan and the UK

Only 4 patients (3%) in Japan and 15 patients (18%) in the UK had both excessive SGB and pathological acid exposure “on” PPIs (AET > 1.6%). In these patients, SGB was not significantly responsible for increased acid exposure in both countries. Of the total AET, 0.6% (0–10.2) (Japan) and 8.8% (0.5–27.0) (UK) was due to SGB-induced acid reflux (*P* = 0.364).

### Impact of Supragastric belching on reflux symptoms in Japan and the UK

Eleven patients (8.9%) in Japan and 16 patients (19.3%) in the UK had both excessive SGB and positive reflux symptom association. SGB was less often associated with reflux symptoms in Japan [0% (0–4.9)] than in the UK [35% (0–54.1), *P* = 0.071] although it did not reach the statistical significance. Six patients (2 Japanese and 4 British) marked reflux symptom within 20 s from SGB not accompanied by reflux. It accounted for 35.1% (17.6–48.2) of the total number of reflux symptoms on average.

## Discussion

Recent studies revealed that SGB is one of the possible mechanisms for PPI refractoriness in patients with GERD symptoms [11, 12]. It is not known whether SGB contributes to PPI-refractoriness similarly in Asia as in Western countries. This study aimed to compare the impact of SGB in PPI-refractory GERD patients between Japan and the UK. All patients underwent impedance-pH monitoring which allows precise phenotyping of GERD as suggested by Lyon consensus and Rome criteria on reflux diagnosis [22, 23]. To our knowledge, this is the first study to assess regional difference of SGB concerning its prevalence and impact on reflux symptoms. We found (1) Japanese patients had almost half as low prevalence of excessive SGB (18%) as the UK (36.1%), (2) SGB had less impact on reflux symptoms in Japan than in the UK although SGB had small impact of AET in both countries.

Impedance-pH monitoring in refractory GERD patients performed “on”-PPI suggested that 33% and 50% of PPI-refractory GERD could be characterized as NERD (> 1.6%) and functional heartburn, respectively [24–27]. Our study shows that Japanese proportion of reflux phenotypes was similar. The British patients, however, showed much higher proportion of NERD patients. This difference might be attributed to the various dose of PPIs regimes. The prevalence of excessive SGB found in British patients studied “on”-PPI (36.1%) was identical to that observed in patients studied “off”-PPI study (35%) [12].

The difference in prevalence of excessive SGB between Japanese and UK patients could not be attributed to clinical or reflux related factors. We found that the geographical difference was the solely factor associated with the different SGB after adjustment for all the different background factors including age, BMI, reflux-related measurements and a various level of gastric acid suppression (i.e., gastric pH < 4 holding time). The Japanese cohort consisted of East Asian racial and ethnic group, whereas, at Royal London Hospital, White, Black or Mixed accounted for roughly two-thirds of the patients and the remaining were south Asians (e.g., Bangladeshi, Pakistani or Indian). Therefore, the “regional difference” can derive largely from the racial/ethnic difference involving different typical diet and cultural background although cross-cultural comparison implies several other potential confounders including health care delivery or referral system [28]. SGB is an acquired behavior which patients unconsciously start to perform to relief an initial unpleasant symptom (e.g., throat, chest or abdominal discomfort) [10, 29]. As we observed no difference in the proportion of patients having chest pain or extra-esophageal symptoms between the two countries, possible different levels of hypervigilance or hypersensitivity among races/ethnics can contribute to different degree of symptom perception and triggering of SGB [15].

Excessive SGB requires dedicated treatment even when not inducing pathological gastroesophageal reflux because SGB itself can cause uncomfortable reflux symptoms. Remarkably, the British patients described more often typical reflux symptoms that were associated with SGB than the Japanese patients although the small sample size could contribute not to reach the statistical significance (*P* = 0.07). Two possibilities might explain the difference. If British patients had more episodes of SGB and reflux symptoms, it may increase the possibility that a SGB is found closer to a reflux symptom just by chance. However, it seems unlikely because patients with excessive amount of SGB had similar number of SGBs in both countries. Alternatively, British patients tend to feel SGB-induced distension of the esophagus, as heartburn more often than Japanese patients. Takeda et al. showed that esophageal balloon distension triggers reflux symptoms (heartburn > chest pain), and the more stretched the esophagus is, the more likely reflux symptoms are triggered [30]. Whether British patients are more sensitive to distension or their SGB involve larger volume of air is unknown. Hypervigilance can also influence the perception of the distension. Hypervigilance is a part of cognitive and affective process which causes patients to pay much more attention to symptoms, and interacts with hypersensitivity mutually [31]. Recent study shows esophageal hypervigilance can predict dysphagia severity better than objective motility-related parameters [32]. Further studies are required to compare the prevalence of esophageal hypervigilance among race/ethnics.

This study has some limitations. First, this was a retrospective analysis of reflux monitoring from patients at tertiary referral hospitals and might not represent the whole GERD population. Second, the dose and types of PPIs/P-CAB were not standardized and third, we could not assess levels of hypersensitivity and hypervigilance in both populations. Lastly, psychological factors were not evaluated by a validated questionnaire although the prevalence of mental disorders was not different between two countries.

In conclusion, the study found that Japanese PPI-refractory GERD patients had lower prevalence of excessive SGB than British patients regardless of reflux phenotype. SGB did not significantly contribute to pathological acid exposure in both countries during on-PPI studies. SGB was more relevant to reflux symptoms in the UK. As the regional difference was the only relevant factor, further studies are required to identify genetic, cultural or diet differences that can influence the impact of SGB in GERD.
